# Exploring the Multifunctionality of Mechanochemically Synthesized γ-Alumina with Incorporated Selected Metal Oxide Species

**DOI:** 10.3390/molecules28052002

**Published:** 2023-02-21

**Authors:** Rabindra Dubadi, Ewelina Weidner, Bogdan Samojeden, Teofil Jesionowski, Filip Ciesielczyk, Songping Huang, Mietek Jaroniec

**Affiliations:** 1 Department of Chemistry and Biochemistry, Kent State University, Kent, OH 44242, USA; 2 Institute of Chemical Technology and Engineering, Faculty of Chemical Technology, Poznan University of Technology, Berdychowo 4, PL-60965 Poznan, Poland; 3 Department of Fuel Technology, Faculty of Energy and Fuels, AGH–University of Science and Technology, Al. A. Mickiewicza 30, PL-30059 Krakow, Poland

**Keywords:** mechanochemical synthesis, γ-alumina, metal oxide incorporation, NO conversion, CO_2_ capture, antimicrobial properties

## Abstract

γ-Alumina with incorporated metal oxide species (including Fe, Cu, Zn, Bi, and Ga) was synthesized by liquid-assisted grinding—mechanochemical synthesis, applying boehmite as the alumina precursor and suitable metal salts. Various contents of metal elements (5 wt.%, 10 wt.%, and 20 wt.%) were used to tune the composition of the resulting hybrid materials. The different milling time was tested to find the most suitable procedure that allowed the preparation of porous alumina incorporated with selected metal oxide species. The block copolymer, Pluronic P123, was used as a pore-generating agent. Commercial γ−alumina (S_BET_ = 96 m^2^·g^−1^), and the sample fabricated after two hours of initial grinding of boehmite (S_BET_ = 266 m^2^·g^−1^), were used as references. Analysis of another sample of γ-alumina prepared within 3 h of one-pot milling revealed a higher surface area (S_BET_ = 320 m^2^·g^−1^) that did not increase with a further increase in the milling time. So, three hours of grinding time were set as optimal for this material. The synthesized samples were characterized by low-temperature N_2_ sorption, TGA/DTG, XRD, TEM, EDX, elemental mapping, and XRF techniques. The higher loading of metal oxide into the alumina structure was confirmed by the higher intensity of the XRF peaks. Samples synthesized with the lowest metal oxide content (5 wt.%) were tested for selective catalytic reduction of NO with NH_3_ (NH_3_-SCR). Among all tested samples, besides pristine Al_2_O_3_ and alumina incorporated with gallium oxide, the increase in reaction temperature accelerated the NO conversion. The highest NO conversion rate was observed for Fe_2_O_3_-incorporated alumina (70%) at 450 °C and CuO-incorporated alumina (71%) at 300 °C. The CO_2_ capture was also studied for synthesized samples and the sample of alumina with incorporated Bi_2_O_3_ (10 wt.%) gave the best result (1.16 mmol·g^−1^) at 25 °C, while alumina alone could adsorb only 0.85 mmol·g^−1^ of CO_2_. Furthermore, the synthesized samples were tested for antimicrobial properties and found to be quite active against Gram-negative bacteria, *P. aeruginosa* (PA). The measured Minimum Inhibitory Concentration (MIC) values for the alumina samples with incorporated Fe, Cu, and Bi oxide (10 wt.%) were found to be 4 µg·mL^−1^, while 8 µg·mL^−1^ was obtained for pure alumina.

## 1. Introduction

The synthesis of mesoporous materials with tailored porosity was significantly expanded after the discovery of ordered mesoporous silica by the Mobil Oil Company in 1992 [1]. Porous materials may possess micropores (sizes below 2 nm), mesopores (sizes between 2 and 50 nm), and/or macropores (sizes above 50 nm) [2]. Non-silica materials such as carbon [3], alumina [4], metal oxides [5], and metal-organic frameworks [6], were also commonly studied. As compared to silica-based materials, porous alumina is more popular in catalysis, mainly in the petrochemical industries [7] because of its unique surface properties (e.g., acidic, and basic sites) and tailorable porosity [7,8]. There are various methods for the synthesis of porous alumina such as sol-gel [9,10], reverse micelle [11], solvothermal [12], microwave-assisted [13], aerosol [14], polymer templating [15], etc., and many of these methods use both cationic and anionic surfactants [16]. The synthesis of porous metal oxide nanomaterials is gaining attention due to their wide applications, and easy merging with silica, alumina, and carbon frameworks. Various attempts have been made for the synthesis of ordered mesoporous γ-alumina using solvent-based methods. Quan et al. synthesized mesoporous γ-alumina via the sol-gel method using a non-ionic block copolymer, aluminum isopropoxide, aluminum nitrate, and organic solvents [7]. This study was further extended by Morris et al. [17] who adopted a one-pot synthesis to incorporate metal oxides in the alumina framework. In their work one-pot synthesis was conducted via self-assembly of the metal precursor and aluminum isopropoxide in the presence of triblock copolymer [17]. The hydrophilic non-ionic surfactant, Pluronic F127 and Pluronic P123 were used together with metal nitrates to study the textural properties of mesoporous γ-alumina [18]. Although popular, these methods use solvents and often harmful chemicals, making them less attractive, at least in terms of the principles of green synthesis. Wet chemical synthesis is a multi-step process, time-consuming, energy-demanding, and may result in generating large amounts of waste [19,20]. To overcome these existing challenges, an environmentally benign, simple, cost-effective, high-yield, and scalable synthesis of porous materials is required [21]. That is why various green procedures were developed [22,23,24,25], such as the mechanochemical synthesis of biomass-derived porous carbons [26,27].

Mechanochemical processing is one of the most promising eco-friendly alternatives for the synthesis of porous nanomaterials [28]. This approach, which leads to a moderate reduction in particle sizes and the formation of micro- up to nano-sized particles [29], differs from the conventional procedures. The International Union of Pure and Applied Chemistry (IUPAC) defines a mechanochemical reaction as “a chemical reaction that is induced by the direct absorption of mechanical energy” [30]. Mechanochemical processing effectively blends precursors to form nanoscale particles and enhances the chemical reactivity of the products. Mechanochemistry is a broader concept than mechanical grinding as it involves the reduction of particle size and simultaneous chemical reactions to form desired products [31,32]. Mechanochemistry has already been used for the synthesis of a wide range of metallic nanoparticles (NPs), e.g., Fe, Cu, Ag, Cd, Zn, Zr, Ti, etc. [33]. Control of particle size can be achieved by adjusting milling time, grinding speed, ball size, and ball-to-mass ratio [34]. During this process, high-energy milling assures large mechanical stress and bond breakage of the reactant particles, resulting in exposure to reactive atomic layers at the interface, which facilitates the formation of a larger number of defects and results in the desired chemical properties [35]. The importance of mechanochemistry for the synthesis of nanoparticles for antimicrobial applications has been presented elsewhere [36].

Stimulated by prior works [37,38] on the mechanochemical synthesis of crystalline γ-alumina in the presence and absence of a few metal salts, the main objectives of this study include: (i) optimization of the soft-templating mechanochemical synthesis of γ-Al_2_O_3_ with incorporated Bi_2_O_3_, Ga_2_O_3_, Fe_2_O_3_, CuO and ZnO, and (ii) their characterization in terms of the chemical composition, surface area and porosity, and (iii) assessment of their adsorption (CO_2_ adsorption), catalytic (selective catalytic reduction of NO) and antimicrobial properties. Although these diverse applications seem to be disconnected, their successful implementation depends on the enlarged specific surface area achieved by well-developed mesoporosity and the properly modulated surface properties accomplished by the incorporation of metal oxide species into γ-Al_2_O_3_. Namely, this study provides extensive experimental data showing a significant impact of metal oxide incorporation on the physicochemical and structural properties of the resulting alumina-based materials. The rationale for selecting γ-alumina as the support for introducing the above-mentioned metal oxides is because of the unique properties of this material, which is widely used as a catalyst and support for various catalysts [8,39], popular adsorbent for gas- and liquid-phase applications, e.g., CO_2_ capture [40], and antimicrobial compound [41]. Additionally, the above-mentioned metal oxide additives are known for their catalytic and antimicrobial properties [39,41].

## 2. Results and Discussion

### 2.1. Basic Information about the Materials Studied

**Figure 1:**
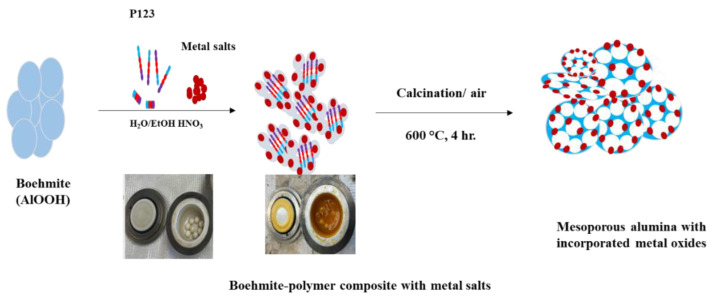
Schematic representation of the synthesis of γ-alumina with incorporated metal oxides.

Schematic representation of the synthesis of γ-alumina with incorporated metal oxides is presented in Figure 1, while the notation of the samples studied with basic information is provided in Table 1.

### 2.2. Compositional Analysis of the Materials Studied

**Figure 2:**
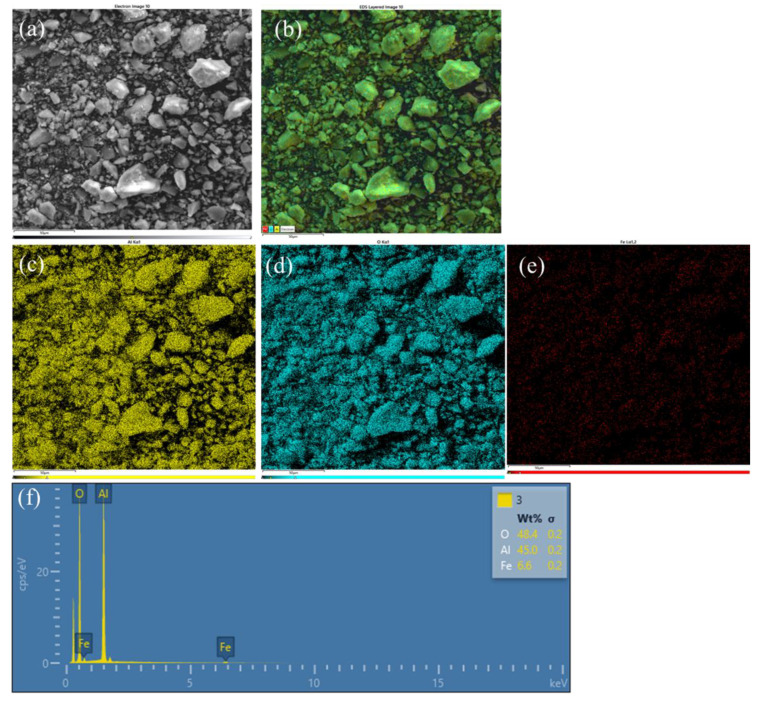
EDX spectrum and elemental mapping for Al-Fe_10_-3: (**a**) SEM image, (**b**) layered elemental mapping, (**c**) aluminum distribution, (**d**) oxygen distribution, (**e**) iron distribution, and (**f**) the corresponding EDX spectrum.

**Figure 3:**
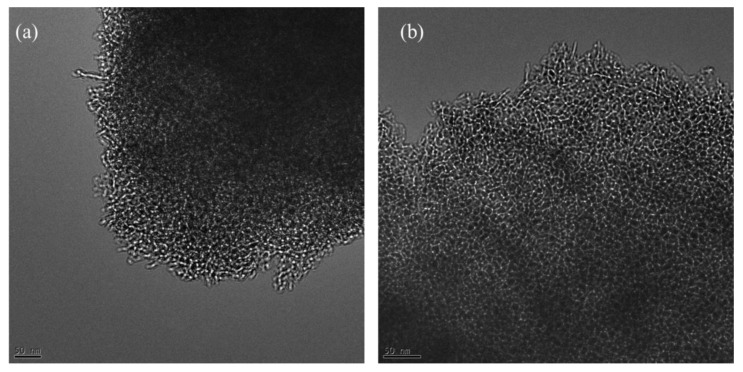
TEM images of Al-Fe_5_-3 (**a**), and Al-Fe_10_-3 (**b**) indicating the presence of disordered but quite uniform mesopores.

The composition of the selected samples was studied using EDX and elemental mapping. The elemental mapping of Al-Fe_10_-3 is displayed in Figure 2 and shows that the iron species are uniformly distributed throughout the sample. Similarly, the elemental mappings of pristine alumina and Al-Cu_10_-3 are shown in Figures S1 and S2, respectively. Additionally, these samples were characterized by TEM to obtain images of Al-Fe_5_-3 and Al-Fe_10_-3 (Figure 3), which show the presence of disordered but quite uniform mesopores. In addition, the TEM images of Al-Cu_10_-3 show a similar distribution of mesopores (Figure S3). The wide-angle X-ray diffraction was also used to elucidate the incorporation of metal oxide species into the alumina framework. The XRD patterns of Al-Fe_5_-3 and Al-Fe_10_-3 (Figure S4) show signals characteristic for γ-alumina with some signs originating from the incorporated iron oxide species as indicated by the spectra of gamma alumina (standard ICSD DB card # 66559) and hematite (standard ICSD DB card # 96075). These tiny XRD signals can be related to the very high dispersion of metal oxide species.

**Figure 4:**
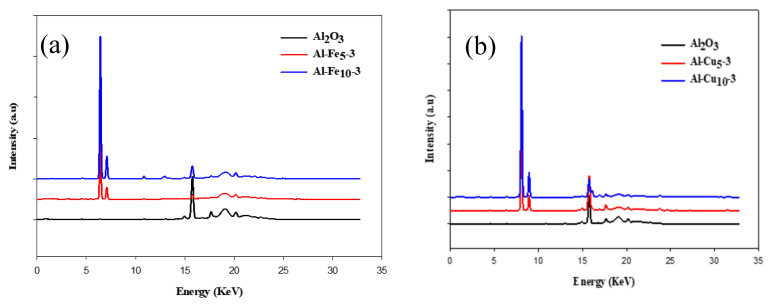

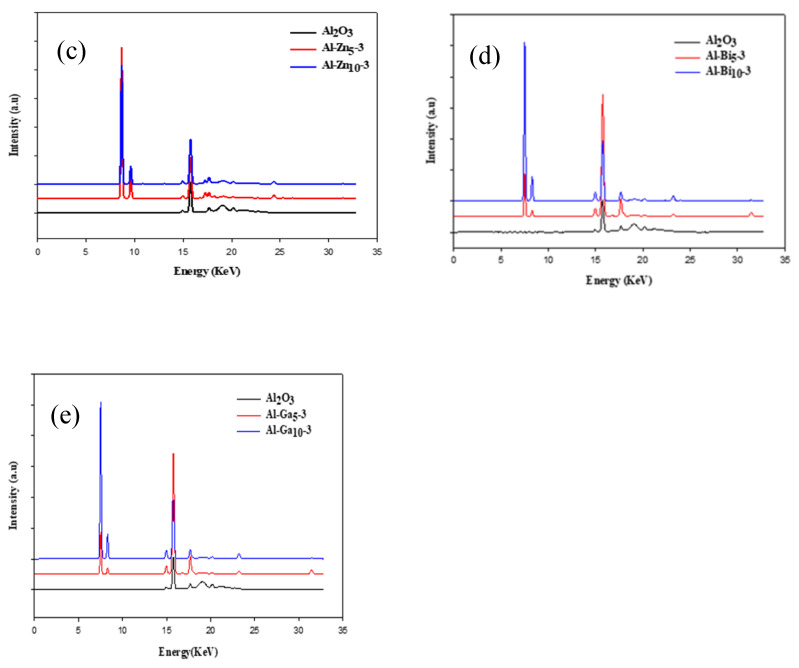
The XRF spectra of Al_2_O_3_ and γ-Al_2_O_3_ samples incorporated with selected metal oxides species: Fe (**a**), Cu (**b**), Zn (**c**), Bi (**d**) and Ga (**e**).

To discover the composition of the obtained materials, an X-ray fluorescence analysis was carried out. The XRF spectra of the samples studied in comparison to the spectrum of pristine Al_2_O_3_ are shown in Figure 4.

All synthesized materials are composed mainly of aluminum oxide as shown in Table 1. The presence of different elements was proven in all samples with incorporated metal oxide species, which indicates the efficiency of the synthesis process. In the case of Al-Fe_5_-3 and Al-Fe_10_-3, signals at 6.40 and 7.06 keV, characteristic of iron were noted. Both materials with incorporated copper oxide show signals around 8.04 and 8.90 keV, characteristic of Cu. For Al-Zn_5_-3 and Al-Zn_10_-3 signals characteristic for zinc are located at 8.63 and 9.57 keV. The alumina with incorporated Ga oxide reveals peaks at 9.24 and 10.26 keV, which confirm the presence of this metal in its structure. Samples Al-Bi_5_-3 and Al-Bi_10_-3 represent peaks at around 10.84 and 13.02 keV, characteristic of bismuth. In all cases, an increased percentage contribution of metal elements in the sample results in increased intensity of XRF patterns, which indicates the effective incorporation of those elements into the alumina structure. The γ-phase of alumina exists at the temperature range of 400–700 °C. The rise in the temperature up to 900 °C transforms the γ-phase to δ-Al_2_O_3_. The thermal behavior of the pristine alumina along with and without triblock copolymer was investigated by a thermal decomposition study as shown in Figure S5. The presence of metal species in the alumina structure changes the decomposition pattern of the triblock copolymer. This behavior agrees with the previous study conducted by Goncalves et al. [39].

### 2.3. Low-Temperature N_2_ Sorption Analysis

**Figure 5:**
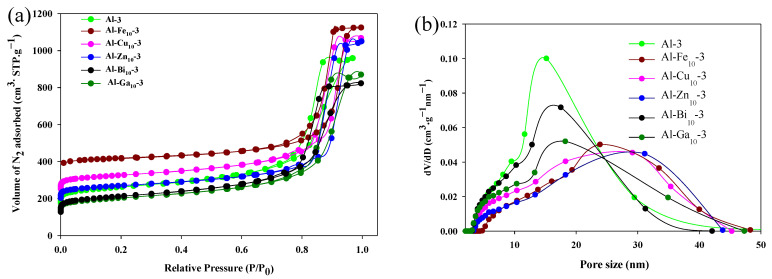
N_2_ adsorption/desorption isotherms for various samples (**a**) with the corresponding pore-size distribution curves (**b**) (obtained from the adsorption branches). For clarity, the isotherms for Al_2_O_3_-3_,_ Al-Fe_10_-3, Al-Cu_10_-3, Al-Zn_10_-3_,_ Al-Bi_10_-3, Al-Ga_10_-3 in (**a**) are offset along the *y*-axis by 175, 350, 250, 200, 225, and 125 cm^3^·g^−1^, respectively.

The textural properties of the mechanochemically synthesized samples were obtained based on low-temperature N_2_ sorption isotherms data. The adsorption isotherms of γ-Al_2_O_3_, the alumina samples with incorporated metal oxide species (10 wt.%) and the reference samples, are shown in Figure 5a together with the corresponding PSD curves. Similarly, the N_2_ adsorption/desorption isotherms and their respective PSD curves of γ-Al_2_O_3_ with 5 wt.% and 20 wt.% metal oxide loading are shown in Figures S6a,b and S7a,b, respectively. The adsorption isotherms obtained for all synthesized samples are of Type IV with the H1 hysteresis loop, characteristic of mesoporous materials [2]. The incorporation of metal oxide species somewhat alters the adsorption isotherms in comparison to those obtained without metal oxides and reference samples. Capillary condensation for all metal-incorporated samples occurs at higher relative pressure because of the presence of larger pores. This feature is validated by the specific surface area, the pore size, as well as the pore volume of the samples studied (Al-Me_5_-3 and Al-Me_10_-3), as shown in Table 2. Data for samples Al-Me_20_-3 are provided in Table S1 and the textural properties for 4, 5 and 10 h milled samples are listed in Table S2.

### 2.4. Catalytic Tests

**Figure 6:**
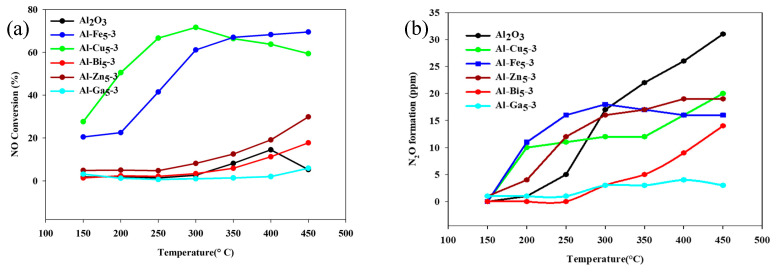
NO conversion (**a**) and the by-product N_2_O formation (**b**) over pristine Al_2_O_3_-3 and different metal oxide incorporated samples (Al-Me_5_-3).

The catalytic performance of the prepared materials was studied for ammonia-induced selective catalytic reduction of NO at the temperature range of 150–450 °C. The results of the NH_3_-SCR catalytic tests obtained for the mechanochemically synthesized materials are presented in Figure 6.

In the case of all tested samples, besides pristine Al_2_O_3_ and gallium oxide-incorporated samples, an increase in the reaction temperature accelerates the NO conversion as shown in Figure 6a. In the case of alumina, after exceeding the temperature of 450 °C, nitrogen oxides are produced rather than NO conversion. This phenomenon is not observed for the metal oxide-incorporated alumina samples. Among all metal oxides used, the copper and iron oxide-incorporated samples showed a significant improvement in the catalytic behavior of alumina. The Al-Cu_5_-3 material reveals the best catalytic properties for the low-temperature SCR, reaching 71% NO reduction at 300 °C. Overall, the highest NO conversion rate was observed for Al-Fe_5_-3 material, reaching a 70% reduction at the temperature of 450 °C. Incorporation of zinc intoAl_2_O_3_ structure caused increased catalytic performance of the material with increasing temperature, but this change is so small, that it is below profitability. Modification of Al_2_O_3_ with bismuth in principle did not affect the catalytic properties of alumina at all and the incorporation of gallium oxide even reduced them.

During experiments, the N_2_O formation, as a by-product, was constantly measured, and the results are presented in Figure 6b. The amount of N_2_O in the case of all samples with incorporated metal oxides was kept low—not exceeding 20 ppm. The highest by-product generation was observed for pristine alumina, which may indicate that metal incorporation in the alumina structure decreases N_2_O production. However, and this is worth mentioning, all the catalytic materials obtained had very low nitrous oxide formation (below 30 ppm). Therefore, it is shown that the effective catalytic reduction of NO is due to the effective incorporation of selected metal oxide species into the alumina structure.

### 2.5. Carbon Dioxide Capture Study

**Figure 7:**
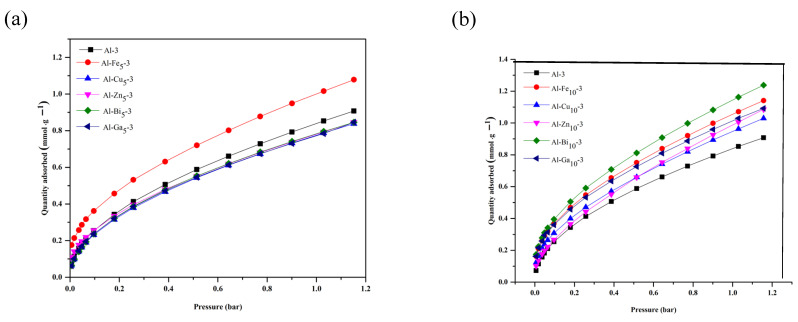
CO_2_ adsorption isotherms measured at 25 °C for pristine alumina and metal oxide-incorporated samples: Al-MeO_5_-3 series (**a**) and Al-MeO_10_-3 series (**b**).

The CO_2_ adsorption isotherms for pristine alumina and all alumina samples with incorporated metal oxides (5 wt.% and 10 wt.%), measured at 25 °C and 1.03 bar pressure, are shown in Figure 7a,b and the amount of CO_2_ captured is listed in Table 1. The general mechanism for CO_2_ capture is based on the interaction between the acidic nature of CO_2_ and the basic site of the metal oxide species in the alumina framework. The development of basic O^2−^ sites on the surface results from high-temperature calcination, which enhances CO_2_ adsorption [42]. During calcination, the surface hydroxyl groups are removed, and some basic sites are formed. The amount of CO_2_ captured by pristine alumina is 0.85 mmol·g^−1^, which is the lowest value compared to that recorded for the alumina samples with incorporated metal oxide species. The observed increase in CO_2_ capture by metal oxide-incorporated alumina samples is due to the presence of additional basic sites exposed on the surface. The highest CO_2_ capture was observed for the alumina samples with incorporated metal oxides (samples obtained by using 10 wt.% of metal). Among them, Al-Bi_10_-3 (1.16 mmol·g^−1^) shows the best adsorption capacity of CO_2_ at 25 °C. The CO_2_ adsorption isotherms and the respective amounts of CO_2_ captured measured for alumina samples containing 20 wt.% of the selected metal oxide are given in Supplementary Materials (see Table S1).

### 2.6. Antimicrobial Activity against Pseudomonas aeruginosa

**Figure 8:**
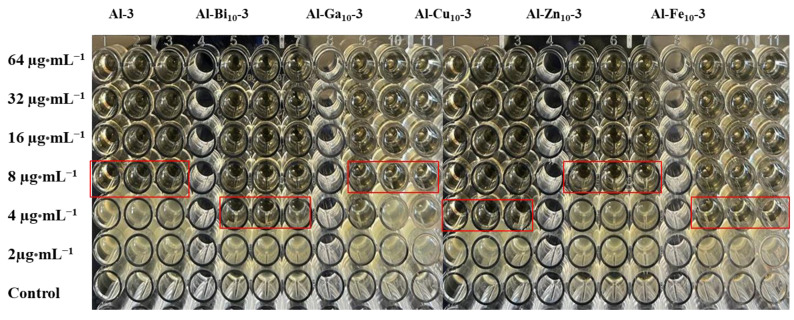
Photographs showing the MIC measurements for Al_2_O_3_, Al-Cu_10_-_3_, Al-Zn_10_-3, Al-Fe_10_-3, Al-Bi_10_-3, and Al-Ga_10_-3 (MIC values are marked in the red box) against *Pseudomonas aeruginosa*.

Samples of alumina with incorporated metal oxide species, synthesized via a one-pot-mechanochemical method, were tested as antimicrobial agents against *Pseudomonas aeruginosa* (PA). The samples calcined at 600 °C were not completely dispersed in the given solvent. Therefore, they were sonicated in DMSO for 10 h and the homogeneous dispersible obtained was used for antimicrobial activity evaluation. PA is a member of the ESKAPE pathogens, a group of Gram-positive and Gram-negative bacteria that can readily evade (i.e., escape) the attack of most clinical antibiotics because of the multidrug resistance (MDR) developed by a variety of phenotypes of these bacteria which can escape the biocidal action of antibiotics and resist their working mechanisms [43,44]. After incubation of PA (ATCC15692) with alumina as a control and metal oxide incorporated alumina for 18 h, the MIC value was found to be 8 µg·mL^−1^ for the control and 4 µg·mL^−1^ for the samples with incorporated Cu (10 wt.%), Fe (10 wt.%) and Bi (10 wt.%) oxides. Similarly, the samples with incorporated Zn (10 wt.%), and Ga (10 wt.%) oxides gave the MIC of 8 µg·mL^−1^ as shown in Figure 8. Higher MIC for the alumina samples with incorporated Zn and Ga oxides might be caused by poor dispersion and agglomeration of the samples. To further explore the biological activity of pristine alumina and metal oxide-incorporated samples, the antimicrobial activity was tested against drug-resistant *Pseudomonas aeruginosa* (DRPA). The activity of pristine alumina (control) was found to be 8 µg·mL^−1^. The antimicrobial activity in the same strain for the samples with incorporated Cu and Fe oxides was found to be 4 µg·mL^−1^ and for those with incorporated Bi, Zn, and Ga oxides, the MIC was found to be 8 µg·mL^−1^. The photographs of the MIC measurements against DRPA for all the samples studied are shown in Figure S9. As expected, the incorporation of 5 wt.% of metal oxide-incorporated alumina structure was found to be less effective than the 10 wt.% contributions, see Figure S10. Surprisingly, alumina samples with the highest metal oxide loading (20 wt.%) were found to be the least effective among all the samples studied. The lower activity might be due to the higher agglomeration of these samples.

**Figure 9:**
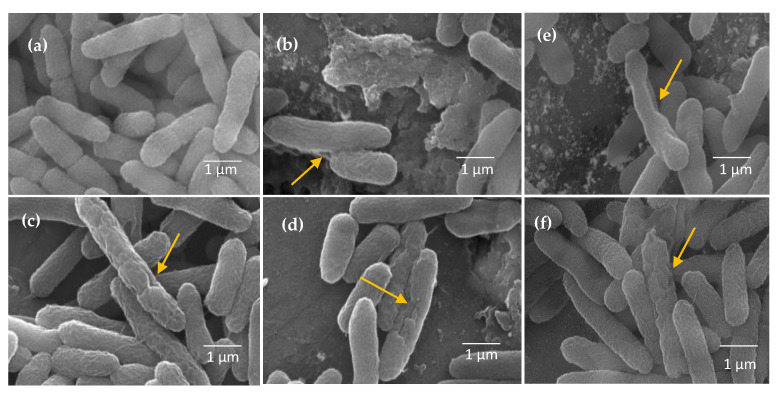
SEM images showing the status of cell morphology and membrane integrity of the bacterial cells treated with Al_2_O_3_ (**a**), Al-Zn_10_-3 (**b**), Al-Fe_10_-3 (**c**), Al-Cu_10_-3 (**d**), Al-Ga_10_-3 (**e**) and Al-Bi_10_-3 (**f**) (yellow arrows show the cell membrane ruptures).

To understand the antimicrobial mechanism of action of the metal oxide-incorporated alumina samples, a morphological study was performed through SEM and the results are shown in Figure 9. As it is shown, the rupture of the cell membrane and release of the intracellular fluid is the main reason for bacteria-killing. In the case of the control sample, the slight deformation and fissures in the cell membrane indicate the cellular activity of the pristine alumina sample. In the case of the samples with incorporated Fe, Cu, Bi, Ga, and Zn oxides, the clear shrinkage and rupture of the cell membrane prove the advantage of metal oxides in the alumina structure, which enhances their biological activity. Thus, this study opens new areas for research concerning mechanochemically synthesized porous samples for biological applications.

## 3. Experimental

### 3.1. Chemicals

All chemicals were used as received without further purification. Catapal A (boehmite), as an alumina precursor was provided by the Sasol Company. Triblock copolymer poly(ethylene oxide)-poly(propylene oxide)-poly(ethylene oxide) Pluronic P123, nitric acid (70%), 200 proof ethanol, metal salts Fe(NO_3_)_3_·9H_2_O, Bi(NO_3_)_3_·5H_2_O, Ga(NO_3_)_3_, Zn(NO_3_)_2_·6H_2_O, and Cu(NO_3_)_2_·6H_2_O, were supplied by Sigma-Aldrich. Deionized water purified by the Milli-Q water purification system was used during the ball milling.

### 3.2. Mechanochemical Synthesis of Metal Oxide-Incorporated γ-Al_2_O_3_

The synthesis of metal oxide-incorporated γ-Al_2_O_3_ was performed using a modified method reported by Szcześniak et al. [37]. Briefly, 1.2 g of boehmite and 3.0 g of P123 were added to 2 mL of deionized water (DI) and 2 mL of 200-proof ethanol. Next, 100 µL of 70% HNO_3_ was added, followed by the addition of the selected metal salt. The control sample was synthesized without the addition of metal salt as depicted in Figure 1. Moreover, the commercial γ-alumina, boehmite, and two-step ground boehmite samples were prepared as a reference for this study. Then, the as-prepared mixture was placed in an yttria-stabilized zirconia (YZrO_2_) grinding jar equipped with eight yttria-stabilized ZrO_2_ balls, 1 cm in diameter each, and milled for 3 h with a rotation speed of 500 rpm using Planetary ball mill (PM200, Retsch). The other milling time (4, 5 and 10 h) was also tested and the selected results are shown in supporting information. The 3 h was found to be the most suitable milling time. Paste-like materials were obtained and furthermore calcined in a quartz glass boat for 4 h, directly in air at 600 °C, at a heating rate of 1 °C/min. This step enabled the removal of the polymer matrix from the final product giving γ-phase alumina, formed at the temperature range of 400–700 °C [39,45] with incorporated selected metal species. The obtained samples were named Al-Me_X_-Y, where Al = Al_2_O_3_, Me = Fe, Cu, Zn, Bi and Ga), x = weight percentage contribution of the metal element (5%,10% and 20%), and Y = the total grinding time (3 h, 4 h and 10 h). The selected samples with their notions are shown in Table 1.

### 3.3. Measurements and Characterizations of γ-Alumina with Incorporated Metal Oxide Species

The X-ray fluorescence (XRF) analysis (Eplison 4, Malvern Instruments Ltd., Malvern, UK) and energy-dispersive X-ray spectroscopy (EDX, PTG Prism Si (Li), Princeton Gamma Tech., Plainsboro, NJ, USA) were carried out to determine the elemental composition of the samples. Wide angle X-ray diffraction (XRD) measurements were collected on a Rigaku Miniflex 600 X-ray diffractometer operating with a Cu anode at a voltage and current of 40 KV and 15 mA, respectively. The scan rate and the step size were 0.25° min^−1^ and 0.02°, respectively, in the range of 10–80°. The XRD spectra were analyzed using PDXL-2 software. A transmission electron microscope (TEM) was operated using FEI Tecnai TF20 FEG TEM at 200 KV equipped with a 4 k ultra-scan charge-coupled device (CCD) camera for high-resolution digital images of alumina samples with incorporated metal oxides.

Nitrogen adsorption-desorption isotherms were measured at −196 °C on ASAP 2010/2020 volumetric adsorption analyzers manufactured by Micromeritics Instruments Co. (Norcross, GA, USA), using 99.998% pure liquid nitrogen. Each sample was degassed under vacuum for at least 2 h at 200 °C before adsorption and CO_2_ sorption measurements. High-resolution thermogravimetric analysis (HR-TGA) experiments were conducted on a TA Instruments TGA Q500 thermogravimetric analyzer. Thermogravimetric profiles were recorded up to 950 °C in flowing air with a heating rate of 10 °C·min^−1^.

### 3.4. Calculations

Brunauer–Emmett–Teller (BET) surface areas (*S*_BET_) were calculated from N_2_ adsorption isotherms in the relative pressure range of 0.05–0.2. Pore size distributions (PSDs) were obtained from the adsorption branch of isotherms using the improved Kruk–Jaroniec–Sayari (KJS) method [46]. Pore widths (*W_KJS_*) were determined from the PSD curves at their apex points. The single-point pore volumes were obtained from the maximum amount adsorbed at a relative pressure of about 0.98.

### 3.5. Selective Catalytic Reduction of NO with Ammonia (NH_3_-SCR)

The catalytic performance of the selected samples was tested in the process of ammonia-induced selective catalytic reduction of NO (NH_3_-SCR), in a fixed-bed flow microreactor under atmospheric pressure in the temperature range of 150–450 °C [47]. To investigate the catalytic properties of the samples, 200 mg of the catalyst was sandwiched between the quartz cotton under flowing He_._ In a typical run, the reaction mixture (800 ppm NO, 800 ppm NH_3_ in He with 3% (*v*/*v*) addition of O_2_) was introduced to the catalytic microreactor through mass flow controllers that maintained the total flow rate of 100 cm^3^·min^−1^. The catalytic unit downstream of the reactor was used to decompose possibly forming NO_2_ to NO [48]. The concentration of residual NO and N_2_O (a by-product of the reaction) in the final stream was measured every 65 s by non-dispersive infrared sensor (NDIR) from Hartmann and Braun. NO conversion was calculated according to the following formula:

*NO conversion* = (*NO*_*in*_ − *NO_out_*)/*NO_in_* (1)

where: *NO_in_*—inlet concentration of NO, *NO_out_*—outlet concentration of NO.

### 3.6. CO_2_ Capture Study

Carbon dioxide adsorption was measured on an ASAP 2020 volumetric adsorption analyzer up to ~1.15 bar pressure and at 25 °C. Each sample was degassed at 200 °C (ramping 1 °C·min^−1^) for 2 h. Then the dewar filled with water at ambient conditions was used to measure CO_2_ capture at that temperature.

### 3.7. Determination of Minimum Inhibitory Concentrations

Guidelines from the Clinical and Laboratory Standards Institute (CLSI) were adopted to determine the Minimum Inhibitory Concentration (MIC) values by the broth microdilution method. The tested bacterial strains of *Pseudomonas aeruginosa* (PA) (ATCC 15692) and drug-resistant PA (DRPA) (ATCC BAA 2108) were cultured [46]. Various concentrations of Al-Me_10_-3 and pristine alumina as a control sample were dispersed in Nutrient Broth (NB) medium with a given strain of bacteria at a density of 1 × 10^6^ CFU·mL^−1^. The resulting suspensions were transferred to a 96-well microtiter plate at 200 μL per well (three wells for each compound). The plate was then incubated at 37 °C for 24 h. MIC values were determined as the lowest concentration that inhibited the visible growth of the tested microorganisms with unaided eyes.

### 3.8. SEM Images of Bacteria

The morphology of incubated PA bacteria was characterized by SEM as previously described [41]. At first, PA bacteria (1 × 10^9^ CFU·mL^−1^) were treated with the control and Al-Me_10_-3 samples, at MIC concentration for 2 h. Similarly, pristine alumina was taken as a control sample with a concentration of 8 μg·mL^−1^. The bacterial solution was then centrifuged at 3750 rpm for 7 min at 4 °C and resuspended in 1 mL of phosphate-buffered saline (PBS) twice. Subsequently, the bacteria were fixed with PBS containing 2.5% of glutaraldehyde. After washing with PBS three times, the bacteria were subjected to a few minutes post-fixation with 1% tannic acid. After fixation, the sample was washed three times with PBS, dehydrated with a series of graded ethanol solutions, dried in air, and coated with gold. SEM images were taken using a Quanta 450 Field Emission Gun Scanning Electron Microscope (FEG SEM).

## 4. Conclusions

The one-pot mechanochemical synthesis of metal oxide-incorporated alumina samples using boehmite as an alumina precursor and liquid-assisted grinding has been shown to be successful and satisfies the main principles of green chemistry and an eco-friendly type of synthesis. The method helps avoid an unnecessary rise in the temperature during friction, shear, or mechanical processing. This method provided pristine alumina with a surface area of 320 m^2^·g^−1^ and a single point pore volume of 0.96 cm^3^·g^−1^. Reference samples such as commercial γ-alumina, boehmite, and two-step synthesized samples show lower surface area and smaller pore volumes. The alumina samples with incorporated metal oxides show somewhat reduced surface area; however, there is a rise in mesoporosity and hence in the total pore volume. The very high total pore volume is an advantageous feature for the selective catalytic reduction of NO. It was found that the catalytic activity of metal oxide-incorporated alumina samples is enhanced in comparison to that of pristine alumina. The iron oxide and copper oxide species incorporated into the alumina structure results in the samples that show the best catalytic properties in SCR (high NO conversion and very low N_2_O formation under a low-temperature range—under 300 °C), among all the samples tested. The materials obtained in this way are potential materials for industrial applications. The formation of γ-alumina was possible by the appropriate thermal treatment at 600 °C of the boehmite-polymer composite. High-temperature calcination facilitates a higher number of basic sites exposed to the surface, and therefore, facilitates a higher CO_2_ capture. The metal oxide-incorporated alumina samples showed an improved CO_2_ capture capacity at ambient temperature compared to that of pristine alumina. The EDX elemental mapping, XRD, TEM, and X-ray fluorescence analysis results confirmed the presence of metal oxides in the samples as evidenced by EDX for Al-Fe_10_-3 and Al-Cu_10_-3 and an increase in the XRF patterns intensity with increasing metal content in each sample. This was further supported by a composition study through XRF. Most of the samples have higher percentages of the respective metal oxides (XRF data) than the predicted percentage based on the amount of metal salt used in the synthesis. The observed difference may be caused due to some losses during sample processing. After proper characterization, the biological activity of the samples was tested, and it was found that analyzed samples are quite active against *Pseudomonas aeruginosa*. The best activity was obtained for Al-Me_10_-3. The Cu, Fe, and Bi samples show better antimicrobial activity than pure alumina. However, the samples with incorporated Zn and Ga oxides exhibit similar MIC values to the control pristine alumina. Similarly, antimicrobial activity against drug-resistant PA (DRPA) was tested and it was found that the samples Al-Cu_10_-3 and Al-Fe_10_-3 show 4 µg·mL^−1^, and the rest of the samples with incorporated metal oxides show 8 µg·mL^−1^.

## Figures and Tables

**Table 1:** Sample notations and metal oxide percentages predicted and obtained by XRF analysis.

Sample	Notation *	Metal Salts Used in the Synthesis	Metal	Added Metal. (Wt.%)	Metal Oxide in the Sample (Wt.%)	XRF Data	
						Al_2_O_3_ (Wt.%)	Metal Oxide (Wt.%)
Al_2_O_3_	Al-3	NA	NA	NA	NA	100.00	NA
Al_2_O_3_-Fe_2_O_3_	Al-Fe_5_-3	Fe(NO_3_)_3_·9H_2_O	Fe	5	3.68	95.42	4.58
Al_2_O_3_-Fe_2_O_3_	Al-Fe_10_-3	Fe(NO_3_)_3_·9H_2_O	Fe	10	7.02	90.69	9.31
Al_2_O_3_-CuO	Al-Cu_5_-3	Cu(NO_3_)_2_·6H_2_O	Cu	5	3.23	96.46	3.54
Al_2_O_3_-CuO	Al-Cu_10_-3	Cu(NO_3_)_2_·6H_2_O	Cu	10	6.22	93.09	6.91
Al_2_O_3_-ZnO	Al-Zn_5_-3	Zn(NO_3_)_2_·6H_2_O	Zn	5	3.19	96.73	3.27
Al_2_O_3_-ZnO	Al-Zn_10_-3	Zn(NO_3_)_2_·6H_2_O	Zn	10	6.16	93.01	6.99
Al_2_O_3_-Bi_2_O_3_	Al-Bi_5_-3	Bi(NO_3_)_3_·5H_2_O	Bi	5	2.87	97.79	2.21
Al_2_O_3_-Bi_2_O_3_	Al-Bi_10_-3	Bi(NO_3_)_3_·5H_2_O	Bi	10	5.57	94.77	5.23
Al_2_O_3_-Ga_2_O_3_	Al-Ga_5_-3	Ga(NO_3_)_3_	Ga	5	3.44	97.74	2.26
Al_2_O_3_-Ga_2_O_3_	Al-Ga_10_-3	Ga(NO_3_)_3_	Ga	10	7.26	94.83	5.17

NA = Not applicable. * Sample notation Al-Me_x_-Y: Al refers to aluminum oxide; Me refers to metal oxide; Y denotes time of ball milling in hours; x indicates Me wt.% equal to x% of Al used in the synthesis (1.2 g of AlO(OH)—boehmite was used in each synthesis, which contains 0.54 g of Al; thus, in the case of Al-Fe_10_-3 in addition of 1.2 g of boehmite containing 0.54 g of aluminum, the specified amount of iron salt containing 10% of Fe equivalent to 10% Al used, i.e., 0.054 g of Fe, was added).

**Table 2:** Textural properties of the synthesized samples.

Sample	S_BET_ (m^2^·g^−1^)	Pore Diameter KJS (nm)	Single Point Pore Volume (cm^3^·g^−1^)	n_CO2_ (25 °C) (mmol·g^−1^)
Commercial γ-Al_2_O_3_	96	34.2	0.41	-
Boehmite	282	1.58	0.34	-
Al-3 *	266	17.7	0.86	-
Al-3	320	15.2	0.96	0.85
Al-Fe_5_-3	307	21.9	1.43	1.02
Al-Fe_10_-3	246	23.9	1.20	1.07
Al-Cu_5_-3	281	17.9	1.21	0.79
Al-Cu_10_-3	277	29.2	1.27	0.96
Al-Zn_5_-3	252	29.3	1.48	0.79
Al-Zn_10_-3	255	31.1	1.32	1.01
Al-Bi_5_-3	300	18.3	1.18	0.80
Al-Bi_10_-3	320	17.5	1.08	1.16
Al-Ga_5_-3	286	18.0	1.15	0.78
Al-Ga_10_-3	280	18.2	1.15	1.03

* Initial grinding of boehmite for two hours, data not available for CO_2_ capture, S_BET_-Specific surface area calculated using the BET equation in the relative pressure range of 0.05–0.20; Single point pore volume obtained from the volume adsorbed at 0.98 P/P_0_; Pore diameter at the maximum of PSD obtained by the KJS method; n_CO_2__–amount of CO_2_ adsorbed at 1.03 bar.

## Data Availability

The data presented in this study are available upon request from the authors.

## Supplementary Materials

The following supporting information can be downloaded at: https://www.mdpi.com/article/10.3390/molecules28052002/s1, Figure S1: EDX spectrum and elemental mapping for γ-Al_2_O_3_ sample; Figure S2: EDX spectrum and elemental mapping for Al-Cu_10_-3 sample; Figure S3: TEM image of Al-Cu_10_-3; Figure S4: Powder XRD patterns of Al-Fe_5_-3 and Al-Fe_10_-3 (b) in comparison with standard spectra for γ-alumina and hematite (Fe_2_O_3_); Figure S5: TGA and DTG profiles of Pluronic (P123), Al_2_O_3_, Al-Fe_5_-3, Al-Fe_10_-3 and Al-Fe_20_-3 before and after calcination; Figure S6: N_2_ adsorption/desorption isotherms and the corresponding pore-size distribution curves for Al_2_O_3_-3, Al-Cu_5_-3, Al-Fe_5_-3, Al-Zn_5_-3, Al-Bi_5_-3, and Al-Ga_5_-3; Figure S7: N_2_ adsorption/desorption isotherms and the corresponding pore-size distribution curves for Al_2_O_3_-3, Al-Cu_20_-3, Al-Fe_20_-3, Al-Zn_20_-3, Al-Bi_20_-3, and Al-Ga_20_-3; Figure S8: CO_2_ adsorption isotherms for pristine alumina and Al-Me_20_-3 (Me = Fe, Cu, Zn, Bi, Ga) samples at 25 °C; Figure S9: Photograph showing the MIC values for Al_2_O_3_, Al-Bi_10_-3, Al-Fe_10_-3, Al-Cu_10_-3, Al-Zn_10_-3 and Al-Ga_10_-3 against drug resistant Pseudomonas aeruginosa (DRPA); Figure S10: Photograph showing the MIC values for Al-Bi_5_-3, Al-Fe_5_-3, Al-Cu_5_-3, Al-Zn_5_-3, and Al-Ga_5_-3, and against Pseudomonas aeruginosa (PA); Table S1: Textural properties of the samples with the highest metal loading; Table S2: Textural properties of the reference samples.
