# Supplementary material for: Exploring the Multifunctionality of Mechanochemically Synthesized γ-Alumina with Incorporated Selected Metal Oxide Species
Source: Molecules. 2023 Feb 21;28(5):2002. doi: 10.3390/molecules28052002 (PMC10004189; doi:10.3390/molecules28052002)
Supplement: Supplementary file 1 [file molecules-28-02002-s001.zip › molecules-2223881-supplementary.pdf]

molecules

MDPI

Exploring the Multifunctionality of Mechanochemically Synthesized γ-Alumina with Incorporated Selected Metal Oxide Species

Rabindra Dubadi [1], Ewelina Weidner [2], Bogdan Samojeden [3], Teofil Jesionowski [2], Filip Ciesielczyk [2], Songping Huang [1] and Mietek Jaroniec [1,*]

[1] Department of Chemistry and Biochemistry, Kent State University, Kent, OH 44242, USA
[2] Institute of Chemical Technology and Engineering, Faculty of Chemical Technology, Poznan University of Technology, Berdychowo 4, PL-60965 Poznan, Poland
[3] Department of Fuel Technology, Faculty of Energy and Fuels, AGH–University of Science and Technology, Al. A. Mickiewicza 30, PL-30059 Krakow, Poland
* Correspondence: jaroniec@kent.edu

Supplementary Materials

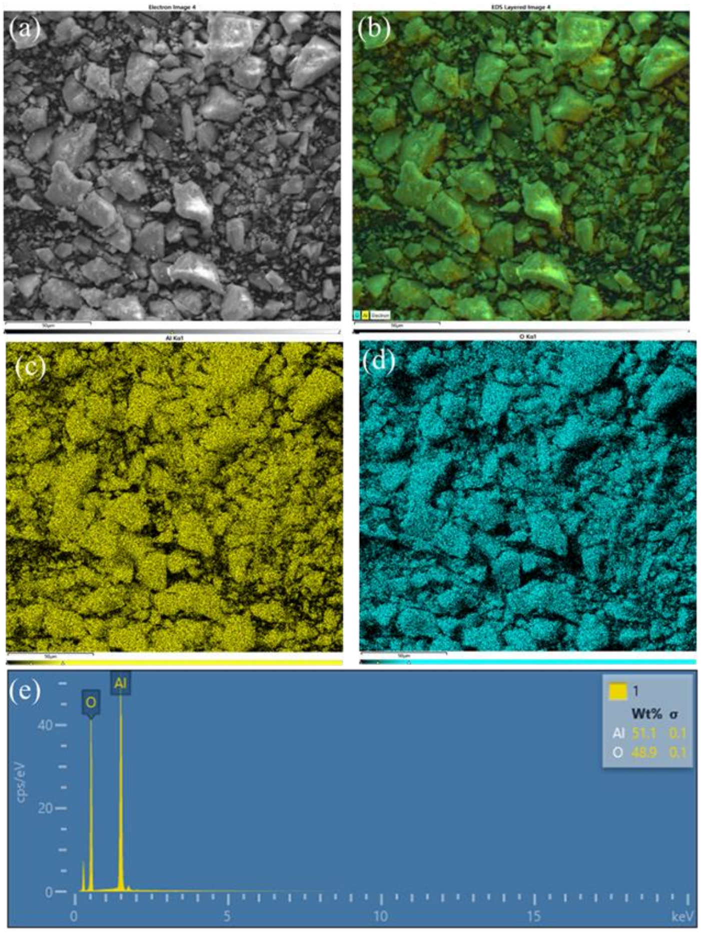

Figure S1. EDX spectrum and elemental mapping for γ-Al_2O_3 sample: (a) SEM image, (b) overlayed elemental mapping, (c) aluminum distribution, (d) oxygen distribution, and (e) the corresponding EDX spectrum.

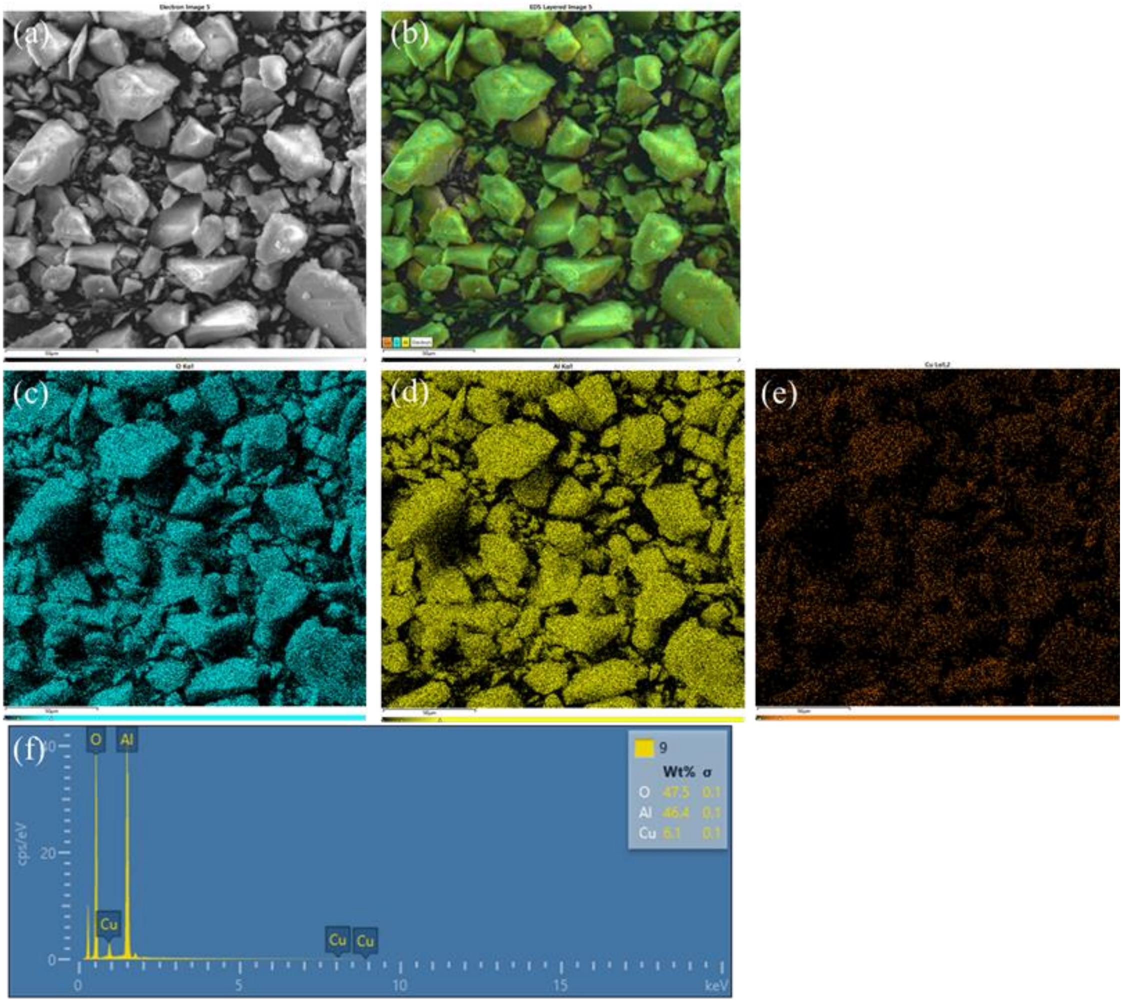

Figure S2. EDX spectrum and elemental mapping for Al-Cu_{10}-3 sample: (a) SEM image, (b) overlayed elemental mapping, (c) aluminum distribution, (d) oxygen distribution, (e) copper distribution, and (f) the corresponding EDX spectrum.

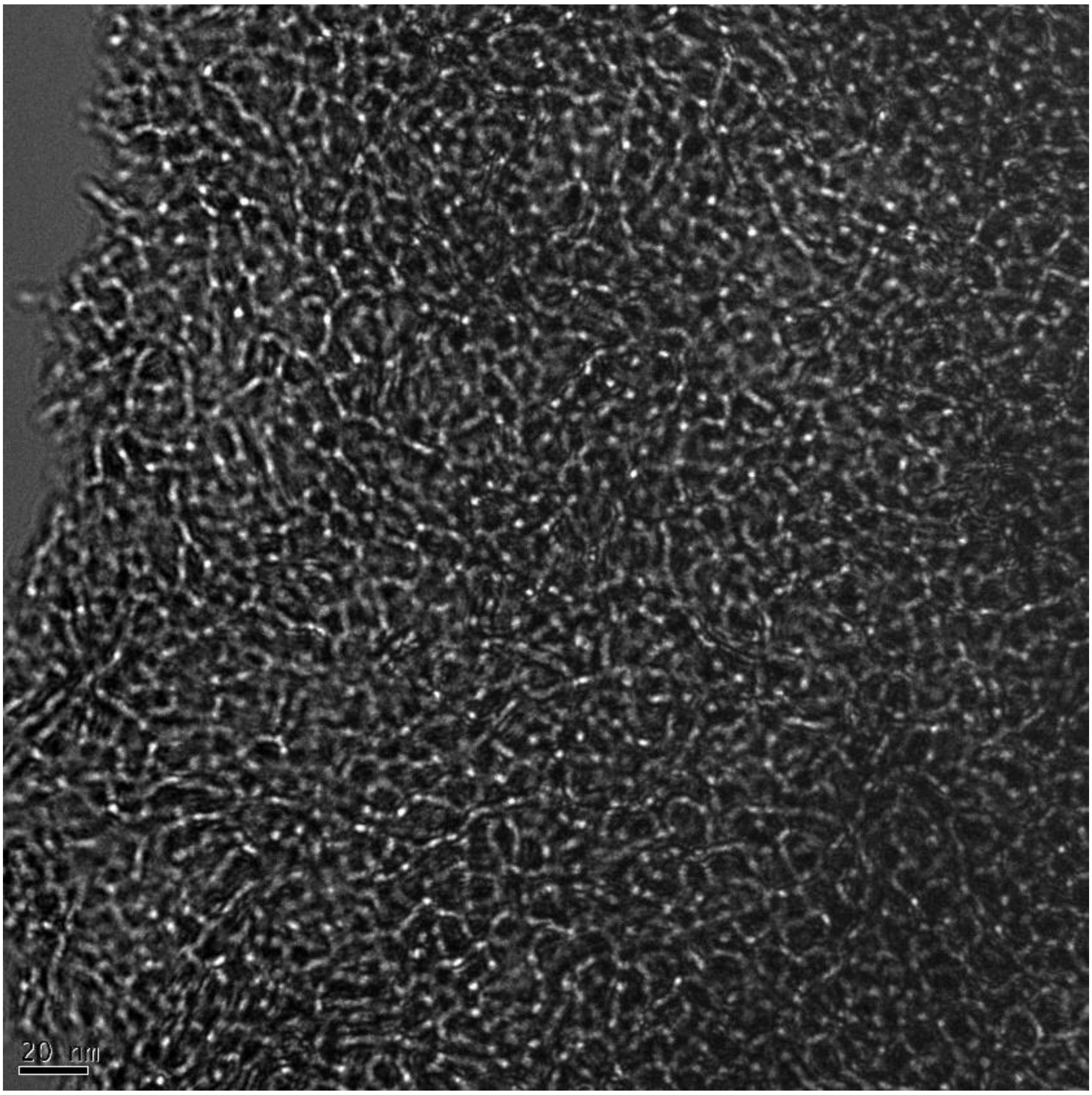

Figure S3. TEM image of Al-Cu_{10}-3 showing the presence of disordered but quite uniform mesopores.

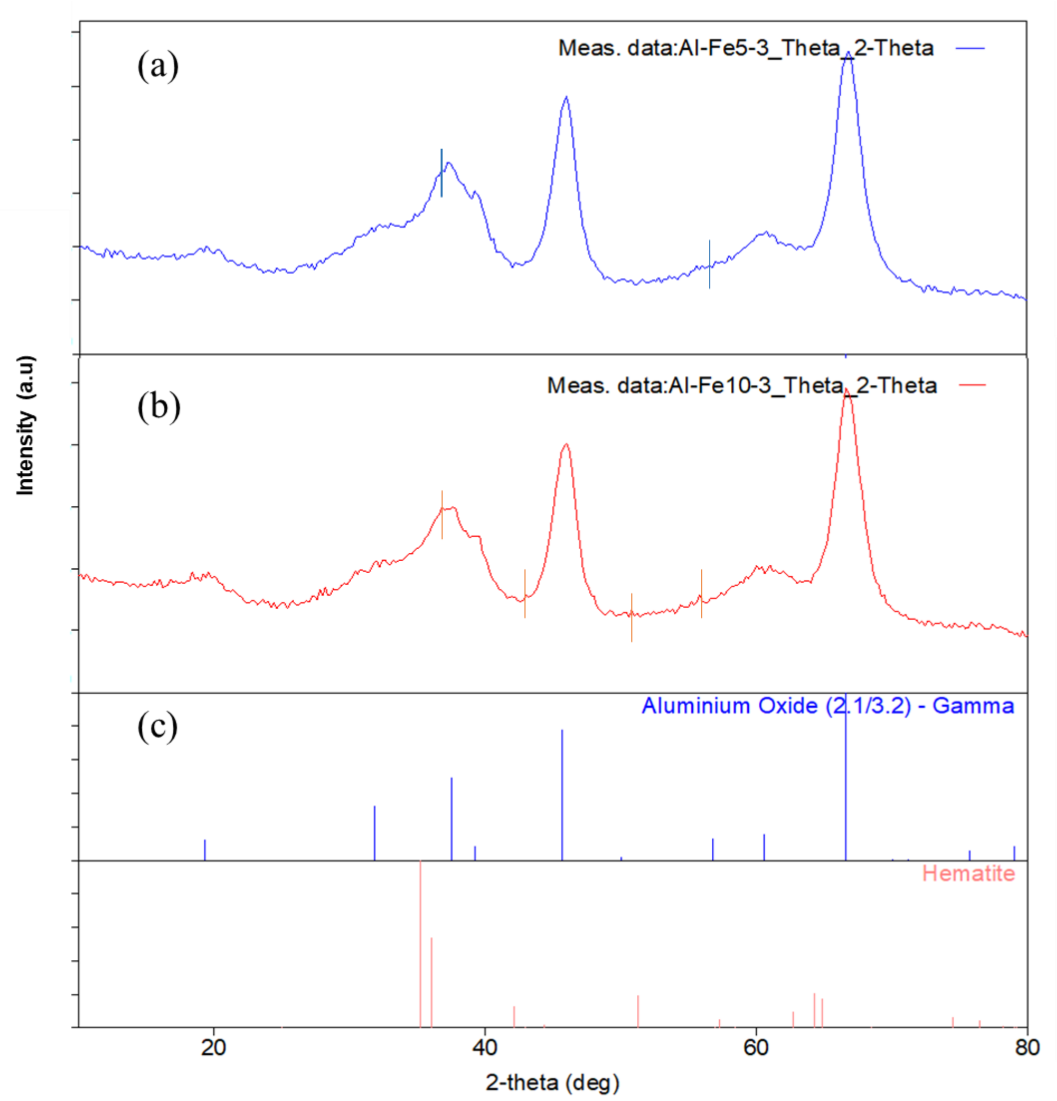

Figure S4. Powder XRD patterns of Al-Fe_5-3 (a) and Al-Fe_{10}-3 (b) in comparison with standard spectra for γ-alumina (c), and hematite (Fe_2O_3) (d).

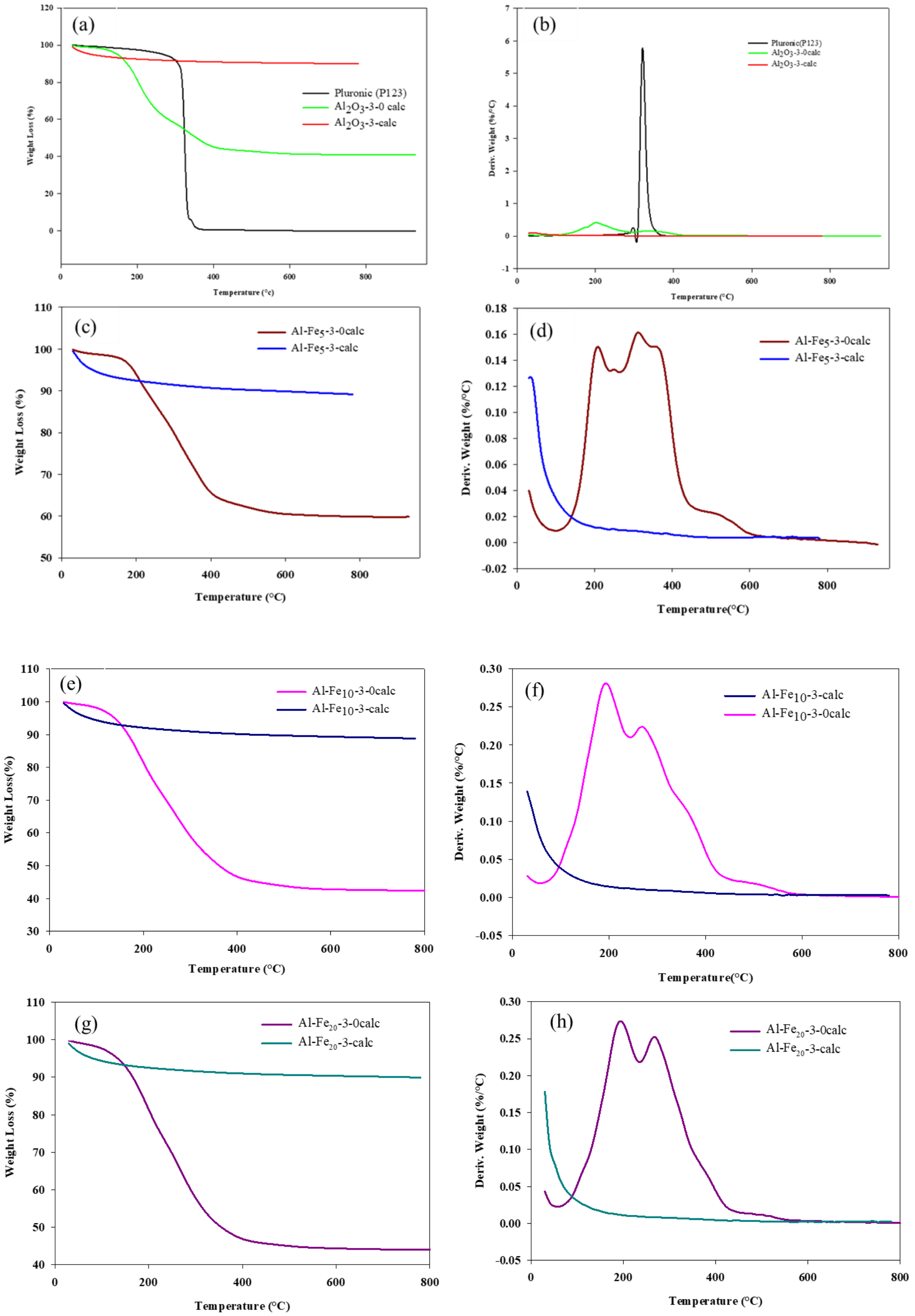

Figure S5. TGA and DTG profiles of Pluronic (P123), Al_2O_3 before and after calcination (a, b), Al-Fe_5-3 before and after calcination (c, d), Al-Fe_{10}-3 before and after calcination (e, f), and Al-Fe_{20}-3 before and after calcination (g, h).

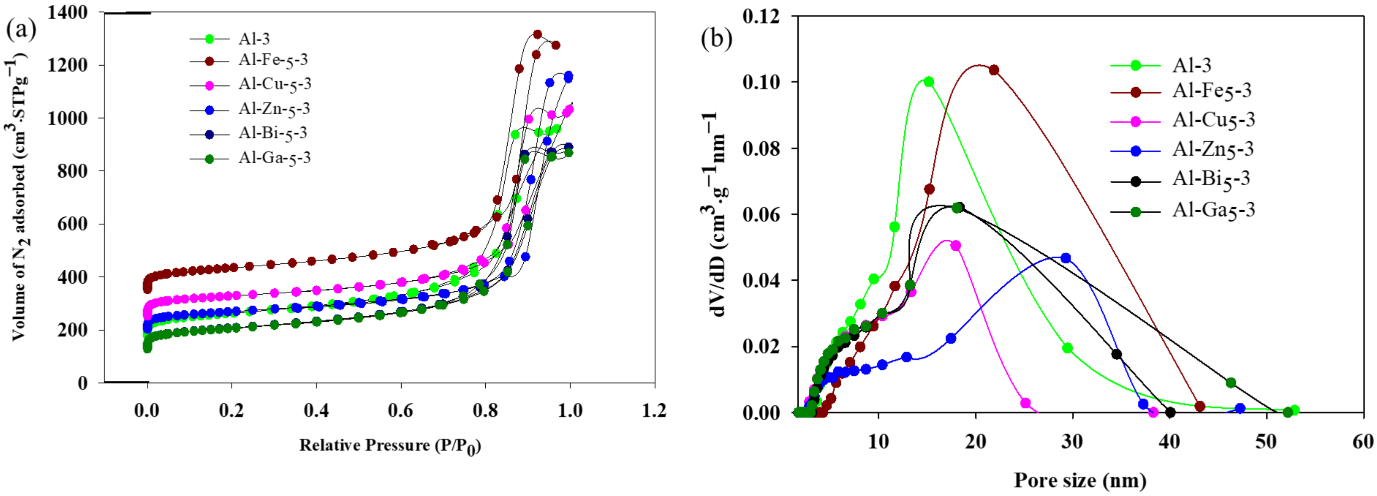

Figure S6. (a) N_2 adsorption/desorption isotherms and (b) the corresponding pore-size distribution curves (obtained from the adsorption branches). For clarity, the isotherms displayed in panel (a) for Al_2O_3-3, Al-Fe_5-3, Al-Cu_5, Al-Zn_5-3, Al-Bi_5-3, and Al-Ga_5-3 are offset along y-axis by 175, 350, 250, 200, 225, and 125 $cm^3{\cdot}g^{-1}$, respectively.

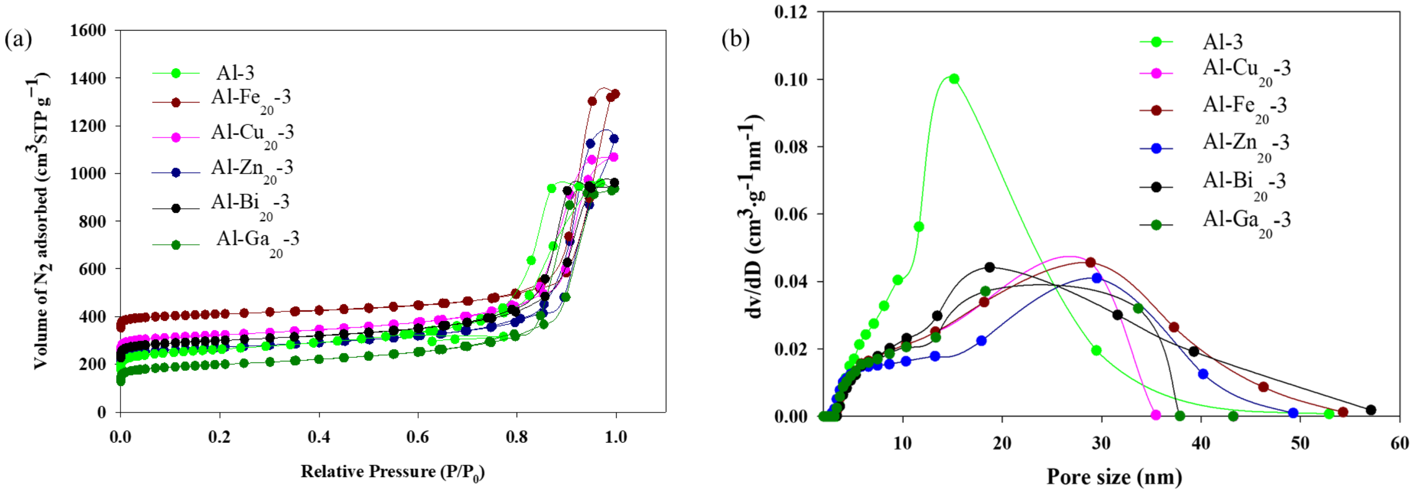

Figure S7. (a) N_2 adsorption/desorption isotherms and (b) the corresponding pore-size distribution curves (obtained from the adsorption branches). For clarity, the isotherms displayed in panel (a) for Al_2O_3-3, Al-Fe_{20}-3, Al-Cu_{20}, Al-Zn_{20}-3, Al-Bi_{20}-3, and Al-Ga_{20}-3 are offset along y-axis by 175, 350, 250, 200, 225, and 125 $cm^3{\cdot}g^{-1}$, respectively.

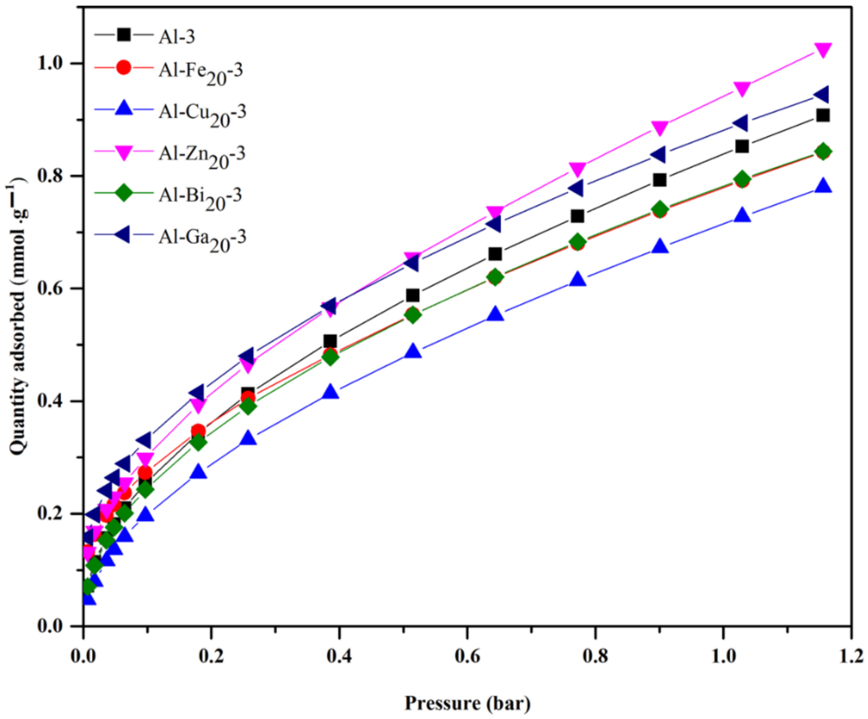

Figure S8. CO_2 adsorption isotherms for pristine alumina and Al-Me_{20}-3 (Me = Fe, Cu, Zn, Bi, Ga) samples at 25 °C.

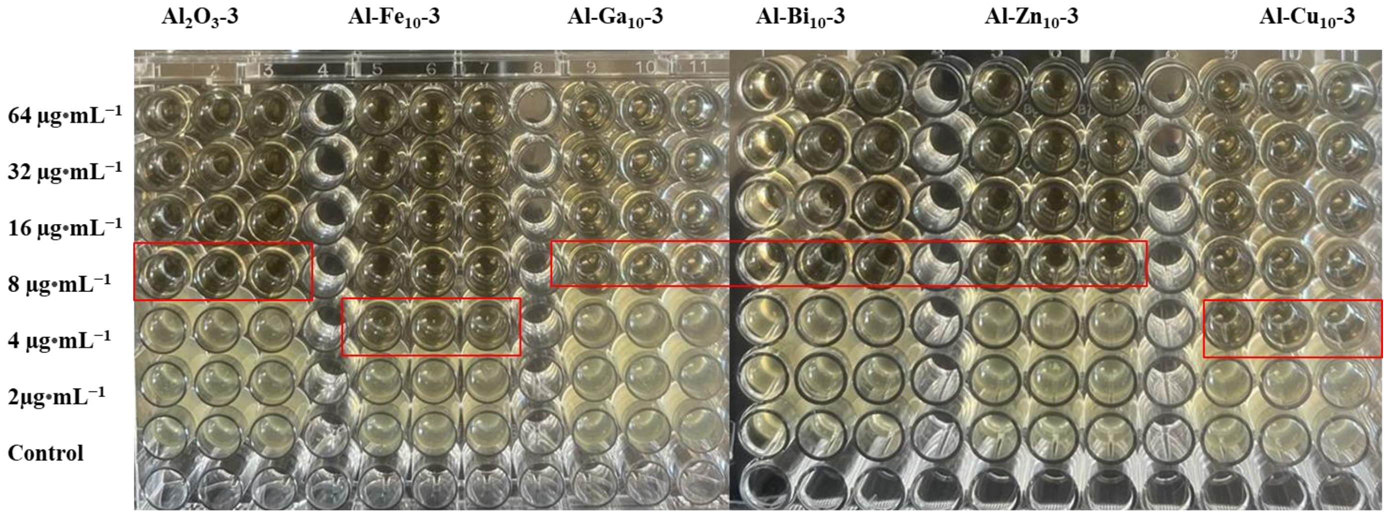

Figure S9. Photograph showing the MIC values for Al_2O_3, Al-Fe_{10}-3, Al-Ga_{10}-3, Al-Bi_{10}-3, Al-Zn_{10}-3, and Al-Cu_{10}-3 (MIC values are marked in red box) against drug resistant *Pseudomonas aeruginosa* (DRPA).

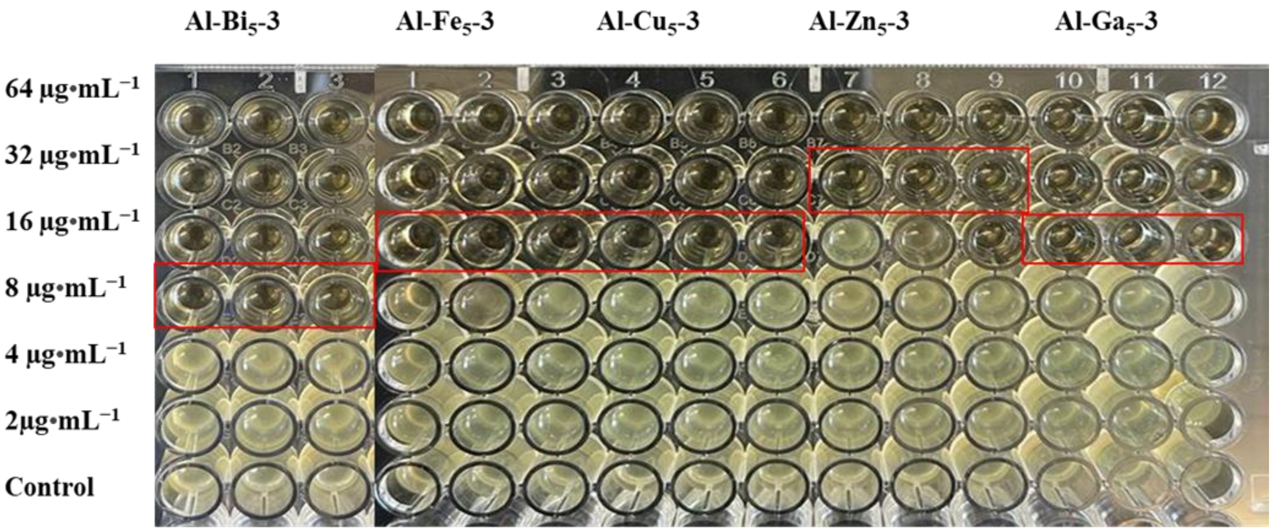

Figure S10. Photograph showing the MIC values for Al-Bi_5-3, Al-Fe_5-3, Al-Cu_5-3, Al-Zn_5-3, and Al-Ga_5-3, and (MIC values are marked in red box) against *Pseudomonas aeruginosa* (PA). The MIC values obtained for alumina samples containing 5 wt.% of metal oxides show the higher antimicrobial activity than samples with 10 wt.% of metal oxides. Due to the agglomeration, the activity of the samples with 20 wt.% of metal oxide is smaller.

Table S1. Textural properties of the samples with the highest metal loading.

**Sample**	**S_{BET} ($m^2 \cdot g^{-1}$)**	**W_{max} KJS (nm)**	**Single point pore volume ($cm^3 \cdot g^{-1}$)**	**n_{CO2} (mmol·g^{-1})**
Al-Fe_{20}-3	218	30.0	1.52	0.79
Al-Cu_{20}-3	263	28.9	1.27	0.73
Al-Bi_{20}-3	269	18.7	1.14	0.79
Al-Zn_{20}-3	257	29.5	1.46	0.96
Al-Ga_{20}-3	268	18.3	1.26	0.89

Note: S_{BET} - Specific surface area calculated using the BET equation in the relative pressure range of 0.05-0.20; Pore diameter at the maximum of PSD curve obtained by the KJS method; Single point pore volume obtained from the volume adsorbed at $0.98P/P_0$; n_{CO2} – amount adsorbed of CO_2 at 1.03 bar.

Table S2. Textural properties of the reference samples.

**Sample**	**S_{BET} ($m^2 \cdot g^{-1}$)**	**W_{max} KJS (nm)**	**Single point pore volume ($cm^3 \cdot g^{-1}$)**
Al-Fe_5-4	291	18.2	1.29
Al-Fe_{10}-4	265	29.5	1.20
Al-Fe_{20}-4	255	28.4	1.45
Al-Fe_5-5	271	18.6	1.16
Al-Fe_{10}-5	270	30.3	1.33
Al-Fe_{20}-5	247	29.4	1.45
Al-Fe_5-10	286	16.1	0.91
Al-Fe_{10}-10	268	18.2	0.98

Note: S_{BET} - Specific surface area calculated using the BET equation in the relative pressure range of 0.05-0.20; Pore diameter at the maximum of the PSD curve obtained by the KJS method; Single point pore volume obtained from the volume adsorbed at $0.98P/P_0$.
